# Long-Term Follow-Up Results of Granulomatous Lymphadenitis Diagnosed by Endobronchial Ultrasound-Guided Fine-Needle Aspiration Biopsy

**DOI:** 10.7759/cureus.34382

**Published:** 2023-01-30

**Authors:** Nevra Gullu Arslan, İlker Yilmam

**Affiliations:** 1 Pulmonary Medicine, Samsun Education and Research Hospital, Samsun, TUR; 2 Pulmonary Medicine, Trakya University Hospital, Edirne, TUR

**Keywords:** long-term follow-up, sarcoid-like reaction, rose, tuberculous lymphadenitis, granulomatous lymphadenitis, ebus-fnab

## Abstract

Introduction

Endobronchial ultrasound-guided fine-needle aspiration biopsy (EBUS-FNAB) is a minimally invasive method used to obtain cytological or histological specimens of masses and lymphadenopathies (LAP) adjacent to the trachea and bronchi. Granulomas, which represent a chronic inflammatory response and occur for a variety of reasons, like a 'sarcoid-like reaction', cause LAPs. In this study, it was aimed to evaluate the long-term follow-up results of granulomatous lymphadenitis diagnosed with EBUS-FNAB and to investigate whether granulomatous lymphadenopathies were precursors of malignancies that occurred during the follow-up period.

Material and methods

The medical records of 123 patients who underwent EBUS-FNAB and were diagnosed with granulomatous lymphadenitis were retrospectively reviewed. Age, gender, acid-fast bacilli (ARB) staining, tuberculosis culture and tuberculosis polymerase chain reaction (PCR) culture results were examined by FNAB, and the procedure indications of all patients diagnosed with granulomatous lymphadenitis were recorded. The long-term health records of 52 patients could not be accessed. Data were collected from 71 patients. The progression, regression or stable conditions of LAPs in the long-term radiological follow-up of at least two years and the treatment conditions of diagnosis after biopsy were examined.

Results

One hundred twenty-three patients were included in the study. Rapid onset evaluation (ROSE) was performed in 93 (75.6%) patients. In 62 (66.6%) of the 93 patients, the smear results were consistent with granulomatous reaction at baseline. Malignancy was present in seven patients (5.6%) at the time of the procedure. In two patients (1.62%), tuberculous lymphadenitis was diagnosed by a positive tuberculosis culture. The long-term follow-up results were not obtained in 52 (42.7%) patients included in the study. At the long-term follow-up of six patients' LAPs with known malignancies, three of them regressed, one of them progressed, and two of them remained stable after chemoradiotherapy. Methylprednisolone treatment was started in eight patients with the diagnosis of sarcoidosis. While LAP remained stable in five patients, regression was observed in three patients. Idiopathic LAPs remained stable in 24 of 55 patients who received no treatment and regressed spontaneously in 31 of them. One of the patients was diagnosed with lymphoma, and the other patient was diagnosed with primary lung cancer in the long-term follow-up.

Conclusion

In cases where tuberculosis is suspected, not only cytomorphology but also microbiological confirmation is important. Granulomatous lymphadenitis can be detected both in the disease course of patients with a history of malignancy and as a precursor to undiagnosed malignancy. So the diagnosis of granulomatous lymphadenitis is a clinicopathological diagnosis that must be followed up in patients without symptoms or other findings.

## Introduction

Lymphadenopathies (LAP), which occur as a result of hyperplasia of lymph nodes for known or unknown reasons, have become a difficult topic to study because they are difficult to access, especially when located in the mediastinum. However, the introduction of endobronchial ultrasound (EBUS) has made it possible to avoid surgical procedures such as mediastinoscopy and video-assisted thoracoscopic surgery (VATS). Endobronchial ultrasound-guided fine-needle aspiration biopsy (EBUS-FNAB) is a minimally invasive method used to obtain cytological or histological specimens of masses and LAPs adjacent to the trachea and main bronchi. In thoracic LAP research, sensitivity and specificity equivalent to mediastinoscopy are reported [1].

While a short diameter of > 10 mm is defined as lymphadenopathy, its causes can be basically divided into malignant and benign causes. Infectious, inflammatory and reactive conditions are the causes of benign LAP. Reactive lymphadenopathy may occur in chronic diseases such as chronic obstructive pulmonary disease, heart failure, bronchiectasis, connective tissue disease and hypothyroidism [2]. Granulomas, which represent a chronic inflammatory response and occur for a variety of reasons, also cause LAP.

On the other hand, the 'sarcoid-like reaction' is a clinical picture that, unlike idiopathic sarcoidosis, occurs as an immunological abnormality in malignant cases and radiologically and histologically resembles thoracic sarcoidosis but without systemic involvement. It is observed in 4.4% of carcinomas, 7.3% of non-Hodgkin's lymphomas and 13.8% of Hodgkin's disease [3]. It may also be observed as a side effect of immunotherapy in this group of patients [4].

In this study, we aimed to evaluate the long-term follow-up results of at least two years in patients who had undergone EBUS-FNAB for mediastinal lymphadenopathy and were diagnosed with granulomatous lymphadenitis on histopathology. Secondly, this study aimed to investigate whether granulomatous lymphadenopathies considered benign at initial diagnosis were precursors of malignancies that occurred during the follow-up period.

## Materials and methods

Ethical approval for this study was obtained under protocol number of SUKAEK-2022 11/18 from the Clinical Research Ethics Committee of Samsun University. The medical records of 123 patients who underwent EBUS-FNAB at the Chest Diseases Clinic of Samsun Education and Research Hospital between 2016 and 2020 and were diagnosed with granulomatous lymphadenitis on histopathology were retrospectively analyzed. The medical records were reviewed using the hospital database and the online system in which physicians can follow health-related information of people in Turkey. Because 52 patients did not have an access to control records or permissions through an online system, the analysis could not be performed. Data were collected from 71 patients.

Age, gender, acid-fast bacilli (ARB) staining, tuberculosis culture and tuberculosis polymerase chain reaction (PCR) culture results examined by needle aspiration for tuberculosis screening and the procedure indications of all patients diagnosed with granulomatous lymphadenitis were recorded. The progression, regression or stable conditions of LAPs in the long-term radiological follow-up of at least two years and the treatment conditions of diagnosis after biopsy in 71 patients were examined.

Statistical analysis

Data obtained from the survey were analyzed using SPSS software (version 26.0, USA) [5]. Descriptive statistics were used to determine the mean and standard deviation (SD) or the number and percentage (%) of variables. Frequency analysis was used for categorical variables. Comparisons between ultrasonographic images of the groups were performed using the chi-square test. The significance level was taken as p < 0.05.

## Results

One hundred twenty-three patients were included in the study; 81 (65.9%) were female and 42 (34.1%) were male. The mean age of the patients was 48.31 years (min-max: 21-82 years). A bedside pathological evaluation (rapid onset evaluation (ROSE)) was performed in 93 (75.6%) patients. In 62 (66.6%) of the 93 patients in whom cell block evaluation revealed a granulomatous reaction and ROSE was performed, the smear results were consistent with a granulomatous reaction at baseline (Table 1).

**Table 1:** Rapid onset evaluation (ROSE) results of the patients ROSE: rapid onset evaluation, NSCLC: non-small cell lung cancer.

	Frequency (n)	Percent (%)
Granulomatous reaction	62	50.4
ROSE not performed	30	24.4
Benign lymphadenopathy	25	20.3
Non-diagnostic	4	3.3
NSCLC	1	0.8
Anthracosis	1	0.8
Total	123	100

Malignancy was present in seven patients (5.6%) at the time of the procedure, including two primary lung cancers and three extrapulmonary malignancies (Table 2).

**Table 2:** Concomitant malignancies in the patients

	Frequency (n)	Percent (%)
No malignancy	116	94.3
Breast cancer	3	2.4
Colon cancer	2	1.6
Lung cancer	2	1.6
Total	123	100

When the ultrasonographic appearance of the lymph nodes of patients diagnosed with granulomatous lymphadenitis was evaluated, the lymph nodes were found to be regularly circumscribed and hypoechoic in 87.8% of cases (Table 3).

**Table 3:** Ultrasonographic appearance of lymph nodes

	Frequency (n)	Percent (%)
Regularly limited hypoechoic	108	87.8
Irregularly limited heterogeneous	7	5.7
Irregularly limited hypoechoic	5	4.1
Regularly limited heterogeneous	3	2.4
Total	123	100

**Figure 1:**
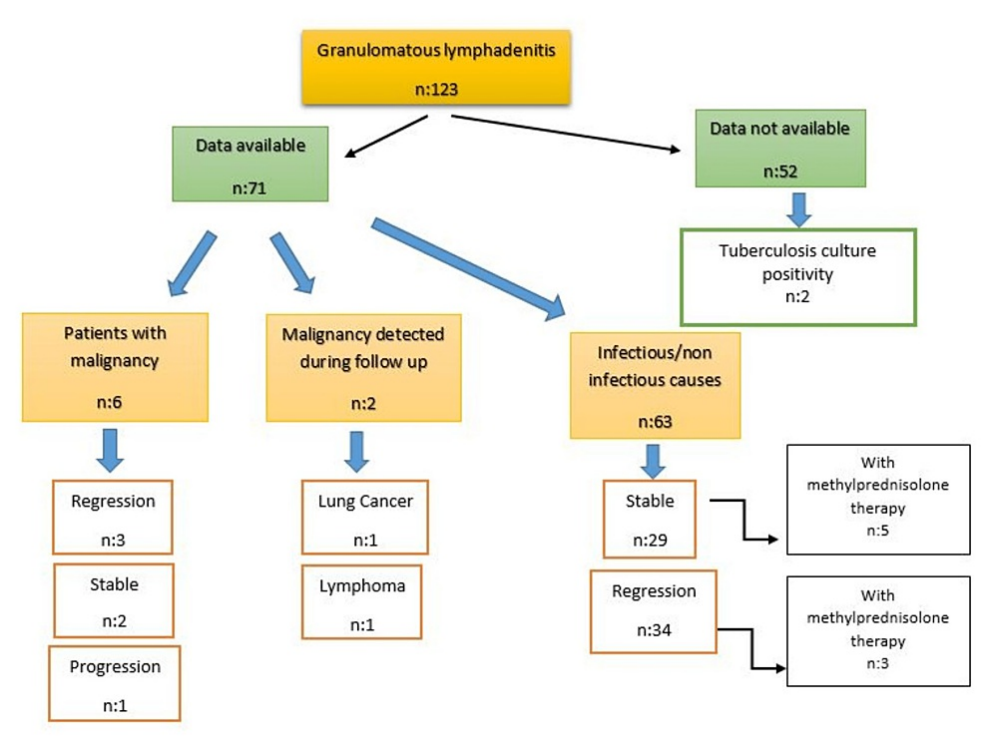
Long-term follow-up, treatment, malignancy association and malignancy development status of patients included in the study

The long-term follow-up results, at least two years, were not obtained in 52 (42.7%) patients included in the study. Of 100 (81.3%) patients, specimens were sent for ARB, tuberculosis culture and PCR through the lymph node. In two patients (1.62%), a positive tuberculosis culture developed and tuberculous lymphadenitis was diagnosed. The long-term follow-up of the patients, concomitant malignancy and treatment status are shown in Table 4 and Figure 1. One of the patients diagnosed with a granulomatous reaction was diagnosed with lymphoma on repeat EBUS examination one year later. The other patient was diagnosed with primary lung cancer in the third year of follow-up.

**Table 4:** Long-term radiological follow-up results of the patients

	Frequency (n)	Percent (%)
Data not available	52	42.27
Regression	37	30.08
Stable	31	25.203
Progression	3	2.43
Total	123	100

## Discussion

Granulomatous lymphadenitis is the result of a chronic inflammatory response triggered by various infectious or non-infectious causes. A variety of diseases such as lymphoma, metastatic carcinoma, sarcoidosis and tuberculosis are involved in mediastinal and hilar lymphadenomegaly etiology. The aim of our study was to investigate the status of LAPs diagnosed with granulomatous lymphadenitis using EBUS-FNAB as a marker for any of the above etiological factors and to determine their changes during long-term follow-up. As a result of this study, it was determined that granulomatous lymphadenitis can occur as a 'sarcoid-like reaction' in malignant patients. Although rare, it may be a precursor to undiagnosed malignant disease. The LAP size may decrease without specific treatment, and granulomas without necrosis do not exclude tuberculosis.

As a result of EBUS-FNAB, the diagnosis of granulomatous lymphadenitis is frequently encountered, and clinicians have difficulty distinguishing it from non-infectious causes. In a study conducted in the United States, the causes of pulmonary granulomatous inflammation were classified as infectious in 54.7% of 226 patients, non-infectious in 26.8% and idiopathic in the remaining 18.4% [6]. In a series of 131 patients in whom granuloma was found after lung resection, infectious causes were found in 49% of patients, non-infectious causes in 11% and no cause in 40% [7]. Similarly, in the 2018 study by Erbay et al., in which they investigated the etiology of granulomatous mediastinal/hilar lymphadenitis, sarcoidosis was observed in 72%, tuberculosis in 3.6% and no cause in 10.9% [8].

In our study, no positive results were observed in any of the 100 patients in whom ARB and tuberculosis PCR were performed from needle aspiration. However, in two patients in whom no necrotizing granuloma was detected, a positive tuberculosis culture developed during follow-up, and treatment was initiated with a diagnosis of tuberculous lymphadenitis. Granulomatous inflammation is common in tuberculosis and sarcoidosis, but the granuloma structure is neither sensitive nor specific. While foci of necrosis are sensitive for diagnosis because of the high cell turnover and macrophage destruction in tuberculosis [9], non-necrotizing granulomas are not specific because they can also occur in tuberculosis. [10]. For this reason, cytomorphology is not sufficient to distinguish between the two diseases in patients who have undergone EBUS-FNAB. It is recommended that microbiology, particularly the presence of necrosis in cytomorphology, and tuberculin-skin test combinations are evaluated together [10].

Non-caseating granulomas, termed 'sarcoid-like reaction', are observed in the lymph nodes of some patients with malignancies and presumably develop the autoinflammatory disease. They are considered paraneoplastic involvement; they may also occur after chemoradiotherapy (CRT) and may mimic metastasis [11]. Six patients in our study had known malignancies at the time of the decision to undergo the EBUS procedure. At long-term follow-up (at least two years, maximum four years) of these LAPs after CRT, regression was observed in three of them, progression in one of them and two of them remained stable. In addition, one patient with no known malignancy at the time of biopsy was diagnosed with lung cancer three years later, and one patient was diagnosed with lymphoma one year later. Therefore, we believe that it should be kept in mind that a 'sarcoid-like reaction' can be found as a precursor finding in an undiagnosed malignant patient, excluding patients with a diagnosis of malignancy, and that intermittent follow-up should be performed.

The incidence of idiopathic granulomatous lymphadenitis varies between 10% and 40% in etiological studies [6-8]. In our study, prednol treatment was started in eight patients who were thought to have sarcoidosis. While LAP remained stable in five patients, regression was observed in three patients. LAPs remained stable in 24 of 55 patients who received no treatment and were classified as idiopathic and regressed spontaneously in 31 of them, and no progressive cases were observed.

In our study, only one (0.8%) of 93 patients who underwent ROSE had a preliminary diagnosis of NSCLC at baseline, but the outcome was reported as granulomatous. In 62 (66.6%) cases, baseline and final outcomes were reported as granulomatous. This high rate supports the view that ROSE is sufficient for the diagnosis of granulomatous lymphadenitis in accordance with the literature [12,13]. The main limitation of our study is the limited number of patients whose data we could access. In addition, detailed information on the dose and duration of treatment was not available for patients treated with methylprednisolone.

## Conclusions

The diagnosis of granulomatous lymphadenitis is a clinicopathological presentation that must be followed up in patients without symptoms or other findings. In cases where tuberculosis is suspected, not only cytomorphology but also microbiological confirmation becomes increasingly important, in countries where tuberculosis is still a common problem. In addition, it should be remembered that granulomatous lymphadenitis can be detected both in the disease course of patients with a history of malignancy and as a precursor to undiagnosed malignancy. We believe that long-term follow-up of more patients, especially those diagnosed with idiopathic granulomatous lymphadenitis, may provide more insightful information on this topic.
